# Exosomal lncRNA TUG1 derived from human urine-derived stem cells attenuates renal ischemia/reperfusion injury by interacting with SRSF1 to regulate ASCL4-mediated ferroptosis

**DOI:** 10.1186/s13287-022-02986-x

**Published:** 2022-07-15

**Authors:** Zejia Sun, Jiyue Wu, Qing Bi, Wei Wang

**Affiliations:** Department of Urology, Beijing Chaoyang Hospital, Capital Medical University, No. 8 Gongti South Road, Chaoyang District, Beijing, 100020 China

**Keywords:** LncRNA TUG1, Urine-derived stem cells, Exosomes, Ferroptosis, Ischemia/reperfusion injury, Acute kidney injury

## Abstract

**Background:**

Human urine-derived stem cells (USCs)-derived exosomes (USC-Exo) could improve kidney ischemia/reperfusion injury (IRI), while the underlying mechanisms of this protective effect remain unclear.

**Methods:**

Human USCs and USC-Exo were isolated and verified by morphology and specific biomarkers. The effects of USC-Exo on ferroptosis and kidney injury were detected in the IRI-induced acute kidney injury (AKI) model in C57BL/6 mice. The effects of USC-Exo on ferroptosis and lncRNA taurine-upregulated gene 1 (TUG1) were detected in hypoxia/reoxygenation (H/R)-treated human proximal tubular epithelial cells (HK-2). The interaction of SRSF1 and TUG1, ACSL4 was checked via RNA pull-down/RIP and RNA stability assays. The effects of LncRNA TUG1 on SRSF1/ACSL4-mediated ferroptosis were verified in H/R-treated HK-2 cells and the IRI-induced AKI mouse models.

**Results:**

USC-Exo treatment improved kidney injury and ameliorated ferroptosis in IRI-induced AKI mouse models. USC-Exo were rich in lncRNA TUG1, which suppressed ferroptosis in HK-2 cells exposed to H/R. Mechanistically, lncRNA TUG1 regulates the stability of ACSL4 mRNA by interacting with RNA-binding protein SRSF1. In addition, SRSF1 upregulation or ACSL4 downregulation partially reversed the protective effect of lncRNA TUG1 on ferroptosis in H/R-treated HK-2 cells. Further, ACSL4 upregulation partially reversed TUG1’s repression on kidney injury and ferroptosis in IRI-induced AKI mice.

**Conclusion:**

Collectively, lncRNA TUG1 carried by USC-Exo regulated ASCL4-mediated ferroptosis by interacting with SRSF1 and then protected IRI-induced AKI. Potentially, USC-Exo rich in lncRNA TUG1 can serve as a promising therapeutic method for IRI-AKI.

## Highlights

- LncRNA TUG1 carried by USC-Exo regulates cell ferroptosis.
- LncRNA TUG1 regulates the stability of ACSL4 mRNA by interacting with SRSF1.
- LncRNA TUG1 alleviates IRI-induced AKI by suppressing ACSL4-mediated ferroptosis.

## Introduction

Ischemic/reperfusion injury (IRI) is one of the common reasons for acute kidney injury (AKI), which is implicated in high morbidity and mortality [1, 2]. Addition to the high mortality rates, patients with AKI have a high risk for progression to chronic kidney disease and hastened the development of end-stage renal disease [3]. Unfortunately, despite recent advances in our understanding of the pathogenesis in IRI-induced AKI, largely therapeutic strategies are often ineffective [3, 4]. Therefore, efforts focused on clarifying pathophysiological mechanisms would be expected to find a new therapy to prevent and treat IRI-induced AKI.

Increasing evidence has been demonstrated that stem cell therapy is a new promising therapy for kidney diseases [5–8]. Human urine-derived stem cells (USCs) have been shown to either attenuate kidney dysfunction or pathological morphology improvement in IRI-induced AKI [9, 10]. Moreover, the patient-derived USCs were identified as a novel biomarker to predict the outcome of the kidney disease [11]. Exosomes are vesicles with a bilayer membrane structure rich in proteins, lipids, miRNAs, lncRNA, and other RNA species [12], carrying biologic signals from one cell type or tissue to another, which are taken up by other cells and participate in regulating the pathological process of diseases [13]. The paracrine mechanism of exosomes is one of the main mechanisms of stem cell therapy for AKI [14, 15]. Exosomes derived from different stem cells have been proved to effectively protect against IRI-induced AKI in vivo and in vitro [6, 9]. Some studies suggest that lncRNA carried by exosomes may have an essential role in the immune response of diseases’ pathology and represent a novel target for IRI-induced AKI. LncRNA taurine-upregulated gene 1 (TUG1) was downregulated in AKI, while upregulated TUG1 presented a protective effect on IRI-induced AKI [16, 17]. However, the mechanism by which USCs-derived exosomes (USC-Exo) protect against IRI-induced AKI is unclear, and whether this protective effect is achieved by lncRNA carried by exosomes has not been investigated.

Recently, ferroptosis was discovered as a new regulated cell death which is dependent on iron and reactive oxygen species [18]. Mis-regulated ferroptosis has been demonstrated in multiple physiological and pathological processes like cancer, tissue injury, T cell immunity, and so on [19]. Acyl-CoA synthetase long-chain family member 4 (ACSL4) is a sensitive monitor of ferroptosis, and an important contributor of ferroptosis [20]. ACSL4 downregulation reduces cell ferroptosis and protects the brain and gut IRI [21, 22]. However, the role of ACSL4-mediated ferroptosis in IRI-induced AKI is not clear.

It was reported that LncRNA could regulate the stability and expression of downstream target mRNA and participate in the disease process by interacting with RNA binding proteins [23]. Serine/arginine splicing factor 1 (SRSF1) is an RNA-binding protein from the SR protein family of splicing regulators, which has been proven to be involved in mRNA metabolism, such as mRNA splicing, stability, and translation, as well as other mRNA-independent processes [24]. Studies have shown that SRSF1 expression is downregulated in myocardial IRI, and its overexpression can inhibit myocardial cell apoptosis [25]. In addition, starBase, an online bioinformatics tool, predicted that RNA-binding protein SRSF1 had a potential binding relationship with lncRNA TUG1 and ACSL4. However, whether lncRNA TUG1 regulates ACSL4 expression by interacting with SRSF1 remains unknown, and the role of this interaction in IRI-induced AKI is not elucidated.

In this study, we investigated the potential protective role of lncRNA TUG1 carried in USC-Exo by using in vivo and in vitro models of IRI-AKI. Based on the above findings, we hypothesized that lncRNA TUG1 carried by USC-Exo may regulate ACSL4-mediated cell ferroptosis by interacting with SRSF1 and then alleviate IRI-AKI.

## Materials and methods

**Fig. 1:**
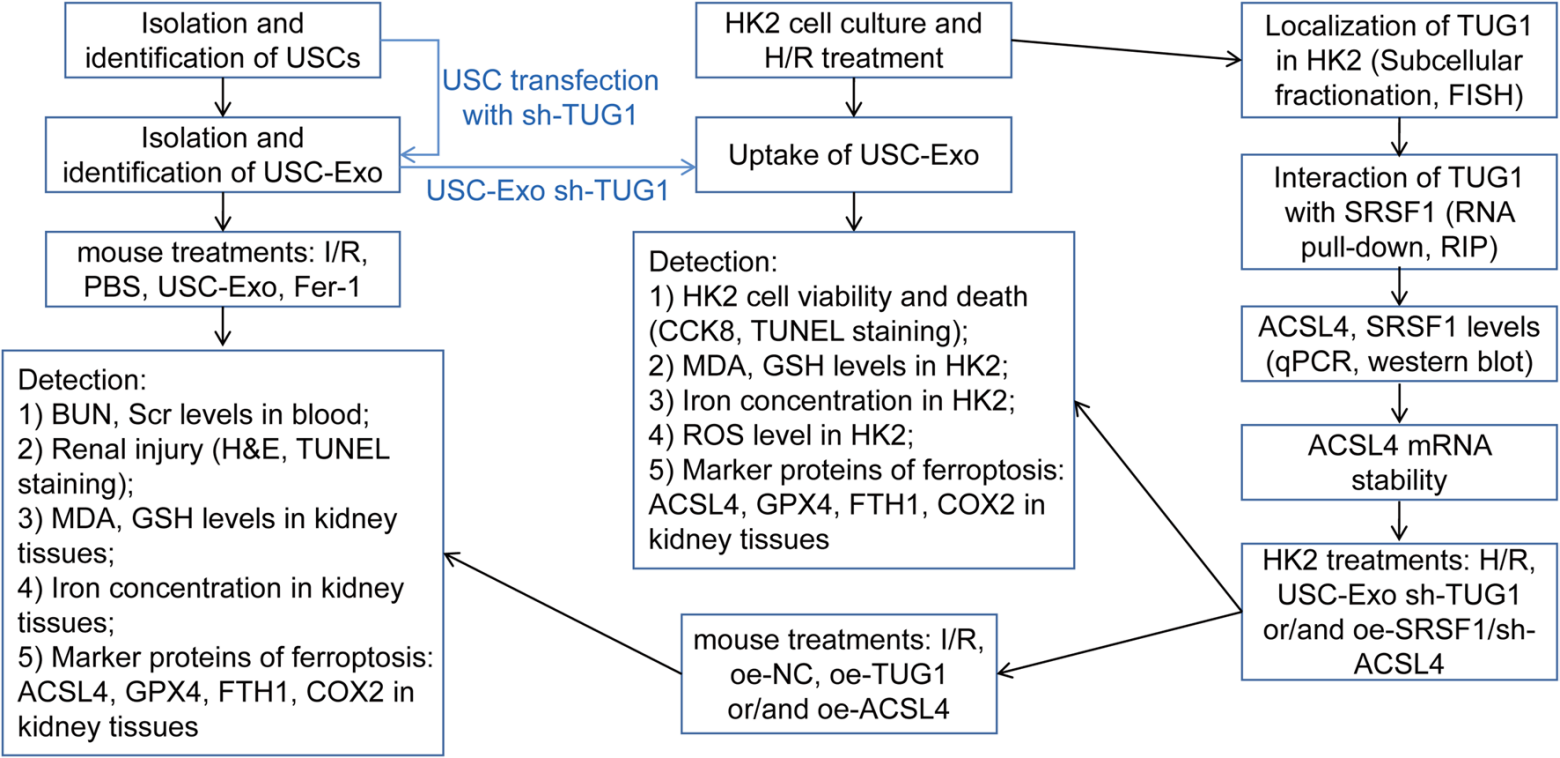
A sequential work flow to illustrate the experimental design. First, we isolated and identified USCs and USC-Exo. To investigate the effect of USC-Exo on the IRI-induced kidney injury and ferroptosis, IRI-AKI mice were treated with USC-Exo or ferroptosis inhibitor Fer-1 or vehicle before IRI 15 min. Next, we explored the functional mechanism in vitro. Whether lncRNA TUG1 carried by USC-Exo affected H/R-induced ferroptosis in HK-2 cells was then determined. In addition, whether lncRNA TUG1 regulated ACSL4 mRNA stability by interacting with SRSF1 was proved. Further, we measured the inhibitory effects of lncRNA TUG1 on ferroptosis through SRSF1/ACSL4 axis in HK-2 cells. Finally, the in vivo impact of lncRNA TUG1 on ferroptosis and kidney injury was assessed via overexpressing TUG1 and ACSL4 in mice

In this study, we first examined the effects of USC-Exo on ferroptosis and IRI-AKI in mice. Then, experiments on the role of USC-Exo and TUG1 suppressing USC-Exo in hypoxia/reoxygenation (H/R)-induced HK2 cells were performed. Besides, mechanism of ferroptosis in HK2 cells was determined. Finally, the regulation of TUG1/ACSL4 axis in ferroptosis was verified in vitro and in vivo (Fig. 1).

### Isolation and identification of USCs

Human USCs isolated from urine samples were collected from healthy male donors (22–28 years old) as previously described [26]. All volunteers signed informed consent before any study, and all experiments of human neutrophils were approved by the Ethics Committee of Beijing Chaoyang Hospital, Capital Medical University (Beijing, China). Urine samples were centrifuged at 400* g* for 10 min at room temperature (RT). After discarding the supernatants, the sediments were resuspended in Dulbecco’s Modified Eagle Medium (DMEM) supplemented with 2% exosome-depleted fetal bovine serum (FBS, Gibco), human epidermal growth factor (hEGF, 10 ng/mL, Gibco), transforming growth factor β (TGF-β, 1 ng/mL, Gibco), basic fibroblast growth factor (bFGF, 2 ng/mL, Gibco), platelet-derived growth factor (PDGF, 2 ng/mL, R&D systems), hydrocortisone (0.5 mM, Sigma), insulin (25 mg/mL, Invitrogen), transferrin (20 mg/mL, Sigma), epinephrine (549 ng/mL, Sigma), triiodothyronine (50 ng/mL), L-glu, and 20 IU/mL of antibiotics. After counting by a hemocytometer, cells were seeded into cellular culture flasks and cultured in a cell incubator at 37 °C for 7 days to allow them to adhere to the substrate. The nonadherent cells were removed by washing with PBS.

USCs were identified by flow cytometry, western blot, and qPCR. USCs were stained with the positive surface antigens antibodies CD29-PE (#303004), CD90-APC (#328114), and CD44-FITC (#397518) and negative surface antigens antibodies CD45-FITC (#304054), CD34-APC (#343510), and HLA-DR-PE (#327008) for 30 min at RT. All antibodies from experiments were purchased from BioLegend (San Diego, CA, USA). Isolated cells were analyzed by flow cytometry using a FACSCanto II (BD Biosciences, San Jose, CA, USA). Isotype antibodies were used as controls. For multilineage differentiation of USCs, cells were treated with osteogenic differentiation-inducing medium (HUXXC-90021, Cyagen Biosciences, Guangzhou, China) or adipogenic differentiation-inducing medium (HUXXC-90031, Cyagen Biosciences) for 2–3 weeks. Then, differentiative capacity was evaluated with Alizarin Red S or Oil Red O staining.

### Isolation and identification of USC-Exo

Human USC-Exo were separated from USCs conditioned medium using total exosome isolation kits according to the company instruction (#4,478,359, Invitrogen). Briefly, at the end time point of culture or treatment according to relevant experiments, the culture medium of USCs was collected by centrifuging at 300*g* for 10 min. Then, the culture media was mixed with exosome isolation reagent and incubated at 4 °C overnight. After incubation, the mixtures were centrifuged at 10,000*g* for 1 h at 4 °C. After discarding the supernatants, exosomes were contained in the pellet of the tube.

For identification, the exosome pellets were fixed with 2% glutaraldehyde for 2 h. After washing, the grids were contrasted in 2% uranyl acetate, dried, and then examined by TEM (Morgagni 268D, Philips, Holland). Exosome's markers and other different specific cell particles surface markers: CD9, CD63, CD81, TSG101, Cytochrome C, Calnexin, and Syntaxin 6, were analyzed by flow cytometry and western blotting to identify USC-Exo.

### Mouse renal I/R model

Mouse renal I/R model was performed in male C57BL/6 mice (8–12 weeks old) as previously described [27]. Briefly, the mice were anesthetized with pentobarbital sodium by intraperitoneal injection and lay on the right side. Dorsal incisions of both left and right sides were made to expose kidneys. The right kidney artery was gently separated with cotton swabs and occluded with a microvascular clamp to induce renal ischemia for 45 min. The left renal pedicle clamping and ischemia were the same as right. After ischemia, the micro-aneurysm clips were removed to start the reperfusion. The wounds were sutured and resuscitated with warm sterile saline intraperitoneally. All operations were the same in the sham group except for clamping and ischemia. All animal experimental protocols were performed according to the guidelines of the Ethical Committee of Beijing Chaoyang Hospital, Capital Medical University.

### Animal treatment

For USCs-Exo treatment experiments, experimental mice were randomly included into following groups: (1) sham (*n* = 6); (2) I/R (*n* = 6); (3) I/R + PBS (*n* = 6), mice underwent renal I/R and injected the same volume (1 mL) of PBS 15 min before the renal artery clamp was placed; (4) renal I/R + USCs-Exo (*n* = 6), mice underwent renal I/R and pre-treated with USCs-Exo (20 μg/mL, 1 mL) intravenously via mouse tail vein 15 min before the renal artery clamp was added; and (5) I/R + ferrostatin-1 (Fer-1, ferroptosis inhibitor, #S7243, selleckchem, German) (*n* = 6), mice underwent renal I/R and pre-treated with Fer-1 (5 mg/kg) intraperitoneally 15 min before the renal artery clamp was placed.

For plasmid treatment experiments, experimental mice were randomly included into following groups: (1) sham (*n* = 6); (2) I/R; (3) For I/R + negative control of overexpression plasmid (oe-NC) group (*n* = 6), mice were pre-treated with the same volume of oe-NC 1 h before underwent renal I/R; (4) renal I/R + oe-TUG1 (*n* = 6), mice were pre-treated oe-TUG1 plasmid (*i.p.*, 60 mg/kg) 1 h before underwent renal I/R; and (5) I/R + oe-TUG1 + oe-ACSL4 (*n* = 6), mice were pre-treated TUG1 and ACSL4 overexpression plasmids (*i.p.*, 60 mg/kg) 1 h before underwent renal I/R. Blood and kidney tissue samples were collected at 24 h post I/R.

### HK2 cell culture and H/R model

HK2 cells purchased from ATCC were cultured in DMEM/Nutrient Mixture F12 supplemented with 10% FBS, 500 U/mL penicillin, and 500 μg/mL streptomycin (Gibco) at 37 °C in a humidified atmosphere containing 5% CO_2_. For H/R treatment, HK2 cells were exposed to hypoxia condition with 1% O_2_, 5% CO_2_, and 94% N_2_ for 24 h followed by reoxygenation (21% O_2_, 5% CO_2_, and 74% N_2_) for 12 h.

### Uptake of exosomes

The PKH67 Green Fluorescent Cell Linker Kit (Sigma-Aldrich) was used to label exosomes. Briefly, USC-exosomes were stained with PKH67 dye for 3 min. After centrifugation, the labeled exosomes were resuspended in PBS. The labeled exosomes were added into the culture medium of HK-2 cells. After incubation for 0, 1, 3 h in the dark, the cells were fixed in 4% paraformaldehyde for 20 min and stained with DAPI. Observing exosomes' uptake was visualized using Zeiss LSM900 confocal microscopy system. The images obtained by confocal microscope were merged and combined by FIJI Image J.

### Cell transfection

Primary USCs or HK-2 cells (from ATCC) were cultured in specific culture medium supplemented with 10% FBS at 37 °C in an incubator. shRNA TUG1 (sh-TUG1, GACTACCTTCCCTGTGCTATT), shRNA ACSL4 (sh-ACSL4, CCAGTGTTGAACTTCTGGAAA), and shRNA negative control (sh-NC) were designed and synthesized by GenePharma (Shanghai, China). For overexpression, TUG1, SRSF1, ACSL4 cDNA were PCR-amplified and then cloned into the expression vector pcDNA3.1 (RiboBio, Guangzhou, China). Lipofectamine 3000 (Invitrogen, Carlsbad, CA, USA) was used for transfection of plasmids into HK2 cells by following the protocol from the manufacturer. Briefly, primary USCs or HK-2 cells were seeded in a 12-well plate (2.5 × 10^5^ cells per well) until the cells are 60–70% confluent. ShRNA was mixed with transfection reagents, and the mixture was incubated for 30 min at room temperature. The cell medium was changed to opti-medium before the transfection. Opti-medium containing the shRNA transfection mixture was added onto the washed cells. The cells were incubated for 6 h at 37 °C in a CO_2_ incubator. After that, normal DMEM medium containing 2 times serum and antibiotics concentration was added without removing the transfection medium. After culturing the cells for additional 6 h, gene expression in cells was checked by qPCR analysis. For experiments in vivo, TUG1 and ACSL4 expression plasmids were packaged with pPACKH1 Lentivector Packaging Plasmid Mix (System Biosciences, Palo Alto, CA, USA).

### Biochemical assays

Commercially available blood urea nitrogen (BUN, #EIABUN, Invitrogen) and serum creatinine (Scr) detection kits (#C011-2-1, Jiancheng Bioengineering Institute, Nanjing, China) were used to measure the levels of biochemical kidney parameters by following the manufacturer's instructions, using an Olympus AU5400 Automatic Biochemical Analyzer (Olympus, Tokyo, Japan).

### Malondialdehyde (MDA) and glutathione (GSH) assay

The levels of MDA and GSH in kidney tissues or HK2 cell lysates were assessed using a Lipid Peroxidation (MDA) Assay Kit (#S0131M, Beyotime Biotechnology, Shanghai, China) and GSH Assay Kit (#S0053, Beyotime Biotechnology) according to the manufacturer's instructions with a microplate fluorometer at 450 and 405 nm, respectively.

### Hematoxylin and eosin (H&E) dye and transferase terminal UTP nick-end labeling (TUNEL) staining

Kidney sections were fixed in 10% buffered formalin and embedded in paraffin, and then, 3 μm sections were stained with H&E for 3 min and visualized under an optical microscope (Olympus Optical, Tokyo, Japan). Tubular injury was scored semi-quantitatively by a blinded observer who examined at least 20 cortical fields (20× magnification) of H&E-stained sections of each kidney from mice. Tubular injury was defined as tubular dilation, tubular atrophy, tubular cast formation, sloughing of tubular epithelial cells or loss of the brush border and thickening of the tubular basement membrane using the following scoring system: Score 0: no tubular injury; Score 1: < 10% of tubules injured; Score 2: 10–25% of tubules injured; Score 3: 25–50% of tubules injured; Score 4: 50–74% of tubules injured; and Score 5: > 75% of tubules injured.

Cell apoptosis of kidney tissue or HK-2 cells was detected by using TUNEL assay (Roche, Basel, Switzerland) according to the manufacturer's instructions. The kidney tissue slides or HK-2 cells were fixed, paraffin-embedded, and labeled with TUNEL reaction mixture containing terminal deoxynucleotidyl transferase and nucleotides including tetramethyl rhodamine-labeled (TMR-labeled) dUTP at RT for 1 h. After staining the nucleus with DAPI, tissues and cells were covered by a fluorescent mounting medium. The TUNEL-positive cells were counted in the five fields of renal cortex region for each section which were counted at 40× magnification, and the percentage of TUNEL-positive cells was calculated from three sections per mice.

### Iron assay

For iron concentration (Fe^2+^ level) detection, HK2 cell or kidney tissue was homogenized using PBS. After centrifugation, iron concentration in the supernatant was assessed by an Iron Assay Kit (#ab83366, Abcam) according to the manufacturer's instructions.

### ROS level measurement

ROS level detection was performed using the ROS Assay Kit (#S0033M, Beyotime Biotechnology) as described in the manufacturer's instructions. In brief, HK2 cells were collected and suspended in diluted DCFH-DA, followed by incubation for 20 min at 37 °C. After washing the cells three times with a serum-free cell culture medium, fluorescence was examined at the excitation/emission 488/525 nm on a fluorescence microplate reader.

### qPCR analysis

Total RNA was extracted from HK2 cells or kidney tissues with TRIzol Reagent (#15596026, Thermo Fisher Scientific). A PrimeScript™ RT Reagent Kit (#RR600A, TaKaRa Bio) was used for RNA reverse transcription. Then, cDNAs were subjected to quantitative real-time PCR using gene-specific primers according to TB Green™ Premix Ex Taq™ (TaKaRa Bio, Inc.) protocols. Human-18S ribosomal mRNA levels or GAPDH was used as internal controls. Data were calculated using the 2^−ΔΔCt^ method [28]. The primers used in this study are presented in Table 1.

**Table 1:** Sequence of primers for qPCR analysis

Gene	Primer sequence
OCT4	Forward: 5′-CTTGAATCCCGAATGGAAAGGG-3′
	Reverse: 5′-GTGTATATCCCAGGGTGATCCTC-3′
NANOG	Forward: 5′-GTCCCAAAGGCAAACAACCC-3′
	Reverse: 5′-ATCCCTGCGTCACACCATTG-3′
TUG1	Forward: 5′-ACGACTGAGCAAGCACTACC-3′
	Reverse: 5′-CTCAGCAATCAGGAGGCACA-3′
SRSF1	Forward: 5′-CCGCAGGGAACAACGATTG-3′
	Reverse: 5′-GCCGTATTTGTAGAACACGTCCT-3′
ACSL4	Forward: 5′-TTGGGCATTCCTCCAAGTAG-3′
	Reverse: 5′-CCTGCAGCCATAGGTAAAGC-3′
GAPDH	Forward: 5′-AGGTCGGAGTCAACGGATTT-3′
	Reverse: 5′-TGACGGTGCCATGGAATTTG-3′

### Western blotting

Primary USCs, HK2 Cells or kidney tissues were lysed in RIPA lysis buffer containing protease inhibitors (Pierce, Protease inhibitor tablet Cat. No. A32963, Thermo Scientific). Whole-cell lysates were centrifuged at 4 °C, 12,000 rpm for 10 min. The supernatants were collected as proteins. Protein concentration in cell lysates was measured using the ABC protein assay kit (Cat. No. 5000002, Bio-Rad). Then, proteins were separated by 10% sodium dodecyl sulfate–polyacrylamide gel electrophoresis and transferred to a polyvinylidene fluoride membrane (Millipore). After blocking, the membrane was incubated with primary antibodies (Table 2) at 4 °C overnight and HRP-conjugated secondary antibody HRP-conjugated secondary antibody (goat anti-rabbit, #A1055, BOSTER Biological Technology, Wuhan, China) at RT for 1 h. The signals were visualized with ECL substrate (Bio-Rad Laboratories, Inc.).

**Table 2:** Primary antibodies used in western blotting assay

Antibody	Company (Cat. No.)	Working dilution
NEPHRIN	Abcam (ab216341)	1:1000
WT-1	Abcam (ab89901)	1:500
CD9	Abcam (ab92726)	1:2000
CD63	Abcam (ab134045)	1:5000
TSG101	Abcam (ab125011)	1:5000
Cytochrome C	Abcam (ab133504)	1:5000
Calnexin	Cell signaling (#2679)	1:1000
Syntaxin 6	Cell signaling (#2869)	1:2000
ACSL4	Abcam (ab155282)	1:20,000
GPX4	Bioss (bs-3884R)	1:1000
FTH1	Cell signaling (#4393)	1:1000
COX2	Cell signaling (#12282)	1:1000
SRSF1	Cell signaling (#14902)	1:1000
GAPDH	Bioss (bs-10900R)	1:1000

### Cell counting kit-8 (CCK8) assay

A CCK8 assay kit (#ab228554, Abcam) was used for the cell viability assay. HK2 cells were seeded in a 96-well plate and co-cultured with PBS, USCs-derived conditioned medium, and exosomes followed exposed to H/R. Then, 10 μL of CCK8 solution was added into each well and incubated for 2 h at RT. The OD value at 450 nm wavelength was determined using by BioTek Synergy Neo2 multi-mode reader (BioTek Instruments, Inc.).

### Subcellular fractionation and fluorescence in situ hybridization (FISH) assay

For subcellular fractionation, cytoplasmic and nuclear RNAs from HK-2 cells were isolated by using NE-PER Reagent (Thermo Scientific). Then, qPCR measurement was performed to assess TUG1 distribution and normalized to GAPDH (cytoplasm) and U1 (nucleus). For FISH assay, End-labeled 6-carboxyfluorescein (FAM) probes were synthesized for TUG1 (Invitrogen). Cells were seeded on coverslips and fixed using 4% formalin. The cells were stained in hybridization buffer (0.7 M NaCl, 0.1 M Tris (pH 8.0), 0.1% SDS, and 10 mM EDTA) containing the probes. Cells were heated at 55 °C for 30 min, mounted using a mounting medium containing DAPI, and were analyzed by fluorescence microscopy (Olympus Optical, Tokyo, Japan).

### RNA immunoprecipitation (RIP)

RIP experiments were performed using a EZMagna RIP RNA-Binding Protein Immunoprecipitation Kit (Millipore). Briefly, HK-2 cells were harvested and lysed in RIP lysis buffer on ice for 30 min. Subsequently, cell exacts were incubated with RIP buffer (containing magnetic beads conjugated to antibodies against SRSF1 or normal anti-rabbit IgG (Millipore)) for 2 h at RT. After washing, beads were resuspended in Trizol and co-precipitated RNAs were isolated for qPCR examination.

### RNA pull-down assay

Biotin-labeled full-length TUG1 RNA probe was prepared with the Biotin RNA Labeling Mix (#11685597910, Roche, Basel, Switzerland). Biotinylated RNAs were was mixed with lysates prepared from HK-2 cells and incubated at 4 °C for 1 h. The mixtures were incubated with washed Streptavidin agarose beads (#S1638, sigma) at 4 °C for 1 h in binding buffer. RNA bound to the probe was eluted, and the retrieved protein was visualized by western blot.

### mRNA stability assay

HK-2 cells were seeded into 6-well plates and transfected TUG1 and/or SRSF1 overexpression plasmid for 48 h, followed by treatment with Actinomycin D (5 μg/mL). At 0, 3, 6 h time points, Trizol regent was added to extract RNA from the cells and ACSL4 mRNA remaining was analyzed via qPCR assay.

### Statistical analysis

All data were represented as the mean ± standard deviation (SD). GraphPad Prism version 8.0 software (GraphPad Software, Inc.) was applied for statistical analyses in all experimental data. Student's *t* test was used for the comparisons of two groups. Comparisons of multiple groups were performed by using one-way ANOVA and Tukey's multiple comparison test. A *P* value < 0.05 was considered to indicate a statistically significant difference between study groups.

## Results

### Human USCs were isolated and identified

**Fig. 2:**
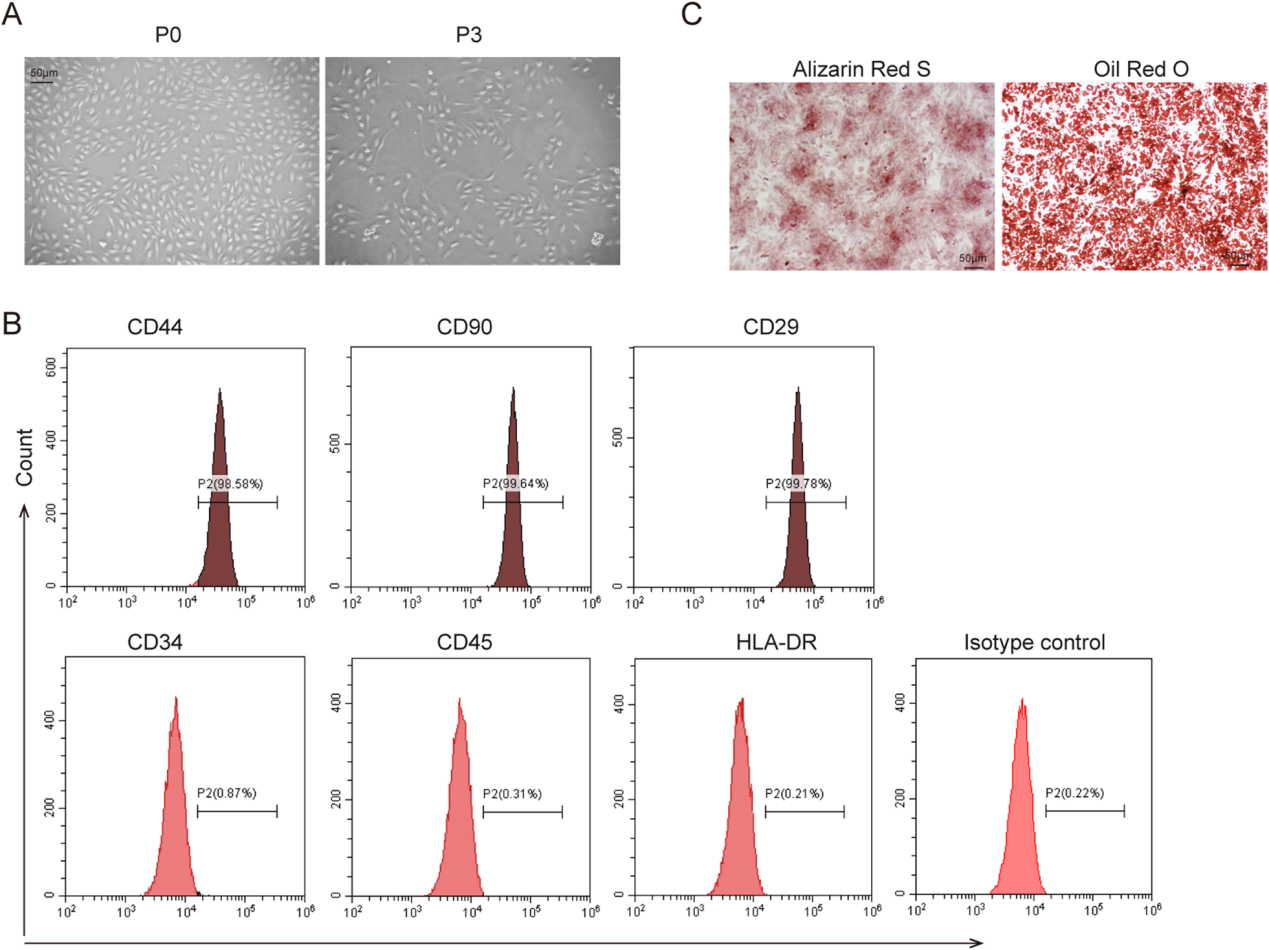
Isolation and Identification of human USC. **A** The morphology of isolated cells was shown by microscope. Scale bar: 50 µm. **B** Isolated cells were stained with surface antigens antibodies and analyzed by flow cytometry. **C** Alizarin Red S and Oil Red O staining after cells were induced. Scale bar: 50 µm. The above data were all measurement data, and all experiment was performed three times

After 3–5 days of primary culture, adherent cells and spindle-shaped colonies were observed. At passage 3, isolated cells showed a spindle-shape and fibroblast-like morphology and rapid proliferation rate **(**Fig. 2A). Human urine-derived cells were analyzed by flow cytometry, which showed that urine-derived cells positively expressed MSCs markers CD44, CD29, and CD90, while negatively expressed hematopoietic antigens CD45, CD34, and HLA-DR (Fig. 2B). In addition, urine-derived cells cultured in an osteogenic medium showed a high osteogenic differentiation ability, whereas Oil Red O staining showed that cells possessed adipogenic differentiation ability (Fig. 2C). Collectively, we successfully isolated human USCs.

### USC-Exo were isolated and identified

**Fig. 3:**
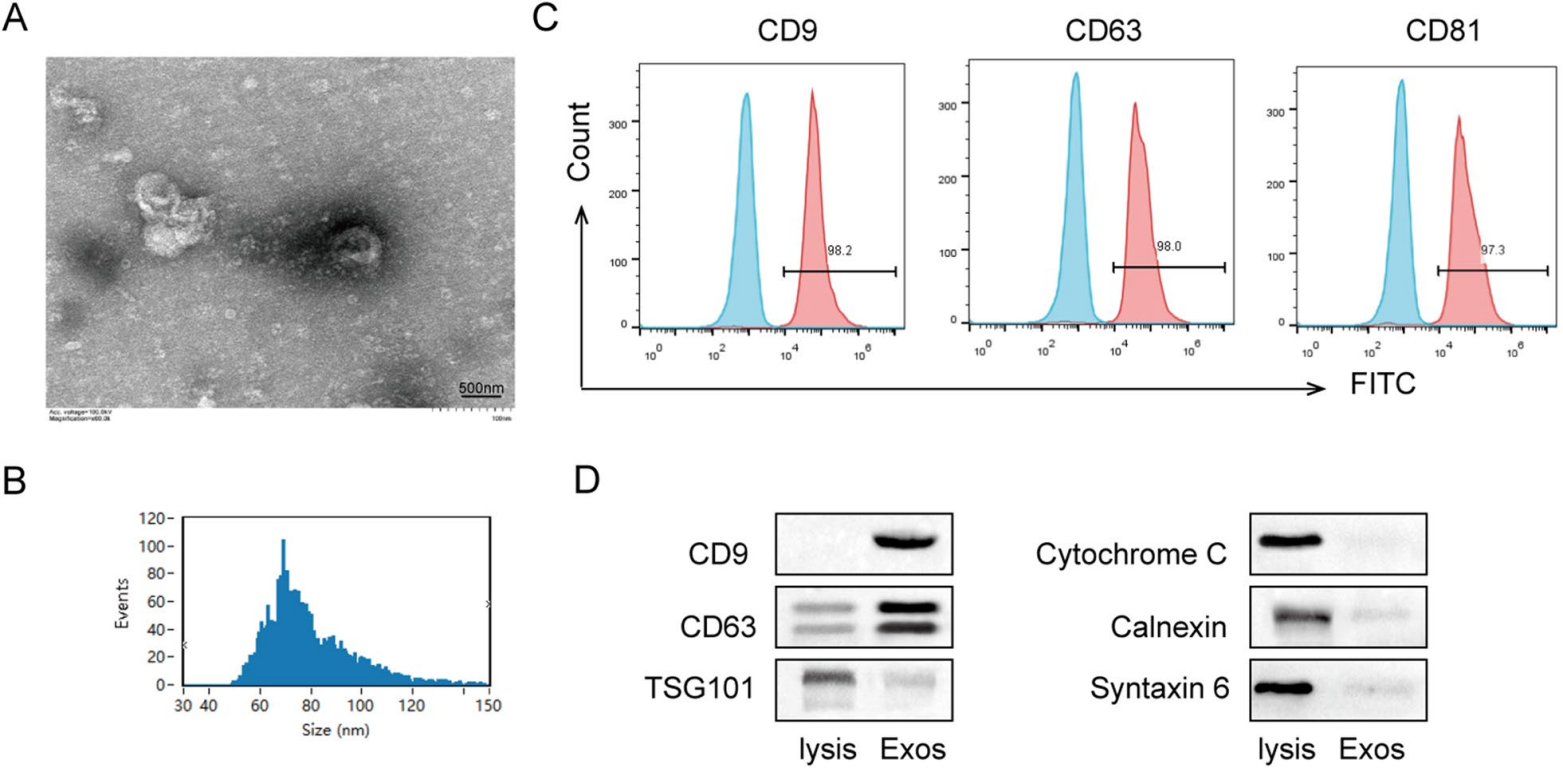
Isolation and identification of USC-Exo. **A** The morphology and size of isolated USC-derived particles were checked by TEM. Scale bar: 500 nm.** B** Nanoparticle tracking analysis of USC-derived particles. **C** USC-Exo were stained with surface antigens antibodies CD9, CD63, and CD81 and analyzed by flow cytometry. **D** The protein expressions of CD9, CD63, TSG101, Cytochrome C, Calnexin, and Syntaxin 6 in USC-Exo were assessed by western blot analysis. The above data were all measurement data, and all experiment was performed three times

TEM, nanoparticle tracking analysis (NTA), flow cytometry, and western blotting were used to identified the USCs-derived cell particles. These particles showed bilayer membrane vesicles the same as exosomes with a diameter of 60–80 nm (Fig. 3A, B). Flow cytometry showed that exosomes markers CD9, CD63, and CD81 were expressed in USCs-derived cell particles (Fig. 3C). Western blot also confirmed that CD9 and CD63 were positive in particles from human USCs, while TSG101, Cytochrome C, Calnexin, and Syntaxin 6 were negative in USCs particles (Fig. 3D). These results suggested that we successfully isolated USC-Exo.

### USC-Exo ameliorate ferroptosis in the kidney after IRI-induced AKI

**Fig. 4:**
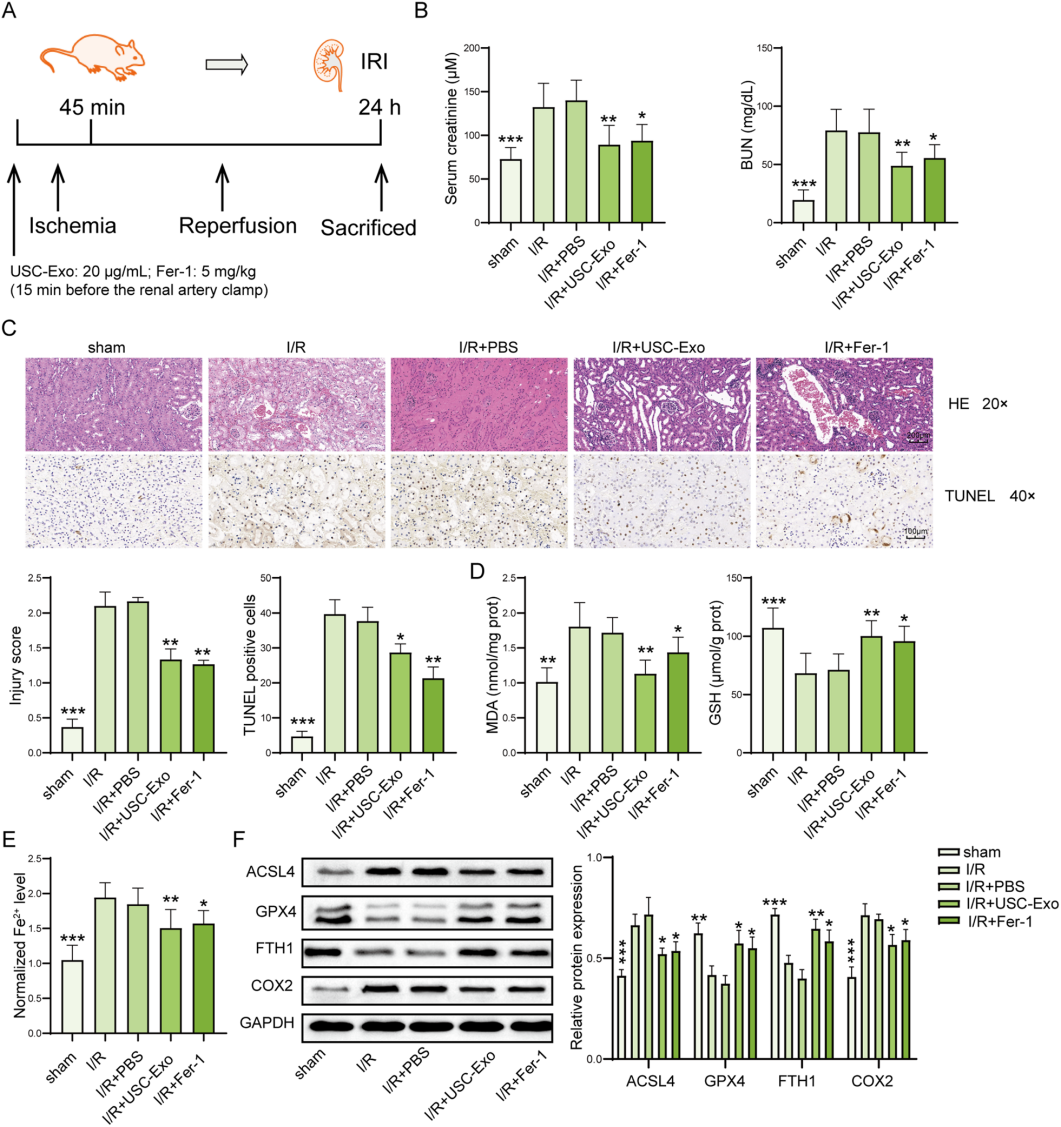
USC-Exo ameliorate ferroptosis in IRI-induced AKI. **A** Diagram of IRI-AKI model and treatment. **B** Serum levels of creatinine and BUN were assessed by colorimetric method. **C** The injury and cell death of kidney tissues showed by H&E dyes and TUNEL assay. Representative images of kidney H&E dyes and TUNEL staining were presented, and the kidney injury score and TUNEL-positive cell quantitative bar diagrams are shown. H&E, scale bar: 200 µm; TUNEL, scale bar: 100 µm. **D** The levels of MDA and GSH in the kidney tissues were measured by commercial kits. **E** The levels of Fe^2+^ in in the kidney tissues of mice were assessed by commercial kits. **F** The protein levels of ACSL4, GPX4, FTH1, and COX2 in the kidney tissues were assessed by western blot and shown as blot image and quantitative bar diagram. *N* = 6 mice per groups. *p < 0.05, **p < 0.01, ***p < 0.001 versus I/R group. The above data were all measurement data and expressed as means ± SD. All experiment was performed three times

To investigate the effect of USC-Exo on the IRI-induced kidney injury and ferroptosis, IRI-AKI mice were treated with USC-Exo or ferroptosis inhibitor Fer-1 or vehicle before IRI 15 min (Fig. 4A). In this work, the serum levels of BUN and creatinine were increased in I/R mice compared to sham group, and this increase was significantly decreased by USC-Exo or Fer-1 treatment (Fig. 4B). The morphological tubular HE and TUNEL dye showed that the kidney tissues of I/R mice were edematous with larger cellular volume, vacuolar degeneration, tubular epithelial cell death and glomerular structure was disordered accompanied by narrowed lumen of the renal tubules. These pathological lesions were alleviated USC-Exo or Fer-1 treatment (Fig. 4C). The tissue levels of MDA and iron were increased, GSH was decreased in I/R mice compared with sham group, and USC-Exo or Fer-1 treatment could partly reverse this trend (Fig. 4D, E). In addition, I/R induced upregulated expression of ACSL4 and COX2, and downregulated expression of GPX4 and FTH1 in kidney tissues, while those effects were improved by USC-Exo or Fer-1 treatment (Fig. 4F). Taken together, USC-Exo suppress kidney cell ferroptosis and have a protective effect on IRI-induced AKI.

### LncRNA TUG1 carried by USC-Exo suppresses H/R-induced ferroptosis in HK-2 cells

**Fig. 5:**
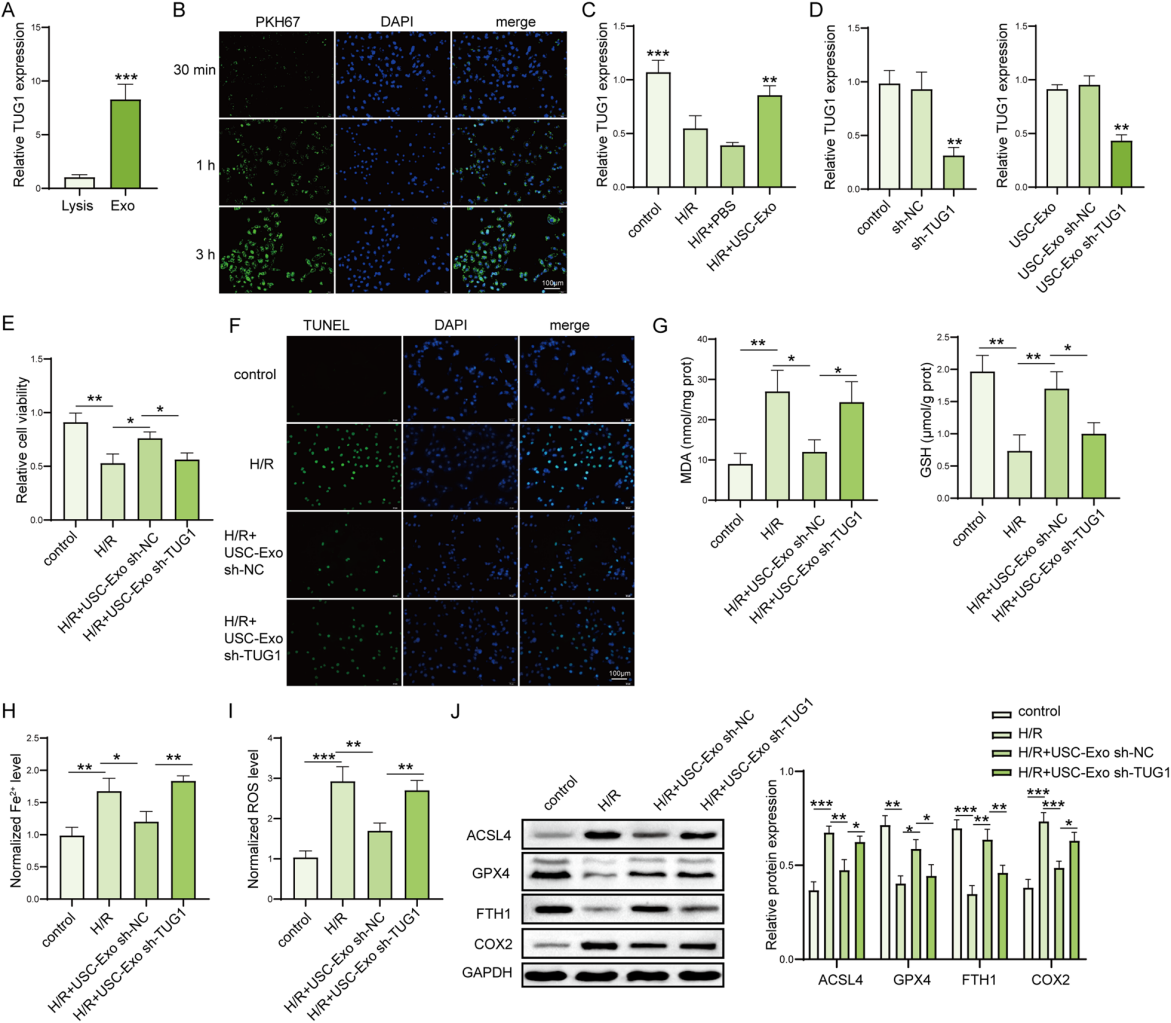
LncRNA TUG1 carried by USC-Exo suppressed H/R-induced ferroptosis in HK-2 cells. **A** The expression of TUG1 in isolated USC-Exo was measured by qPCR. **B** The uptake of USC-Exo by HK-2 cells was checked by PKH67 immunofluorescence dyes. Scale bar: 100 µm. **C** The effect of USC-Exo on the expression of TUG1 in HK-2 cells was detected by qPCR. **D** The expression of TUG1 in USCs (untreated, transfection with sh-NC and sh-TUG1): USC-Exo, sh-NC-treated USC-Exo, and TUG1-silenced USC-Exo, was checked by qPCR. NC: negative control. **E** Cell viability in HK-2 cells after treated with H/R and sh-NC-treated USC-Exo and TUG1-silenced USC-Exo was measured by CCK-8 kits. **F** Cell death in HK-2 cells was checked by TUNEL assay. TUNEL-positive cell quantitative bar diagrams are shown. Scale bar: 100 µm. **G** The levels of MDA and GSH in HK-2 cells were assessed by using commercial kits. **H** The levels of Fe^2+^ in HK-2 cells were assessed by using commercial kits. **I** The levels of ROS in HK-2 cells were detected using DCFH-DA probe. **J** The protein levels of ACSL4, GPX4, FTH1, and COX2 in HK-2 cells were detected by western blot and shown as blot image and quantitative bar diagram. *N* = 3 samples per groups. *p < 0.05, **p < 0.01, ***p < 0.001. The above data were all measurement data and expressed as means ± SD. All experiment was performed three times

Next, the expression of lncRNA TUG1 was significantly increased in the isolated USC-Exo compared with whole cell lysis (Fig. 5A). PKH67 and DAPI dye was used to label exosomes and fluorescence microscopy observed that exosomes can be taken up by HK-2 cells (Fig. 5B). H/R decreased lncRNA TUG1 expression in HK-2 cells compared with control group, while USC-Exo reversed TUG1 expression in HK-2 cells after H/R (Fig. 5C). To verify the role of lncRNA TUG1 carried by USC-Exo in ferroptotic protection, HK-2 cells exposed to H/R were treated with or without USC-Exo, and we adopted siRNA tool to transiently knockdown TUG1 expression in USC-Exo. We found that exosomes from USCs treated with sh-TUG1 significantly decreased the expression of TUG1 compared to exosomes from USCs treated with sh-NC (Fig. 5D). Moreover, H/R significantly decreased cell viability and increased cell apoptotic rate in HK-2 cells, and these effects were partially reversed by USC-Exo, while Exo isolated from USCs transfected with TUG1 shRNA reduced the increase in cell viability and decrease in apoptosis caused by USC-Exo (Fig. 5E, F). USC-Exo inhibited the increased effect on the levels of MDA, iron, ROS, and decreased effect on GSH level by H/R in HK-2 cells, while TUG1 silenced USC-Exo could suppress these changes caused by USC-Exo (Fig. 5G–I). In addition, USC-Exo suppressed the increased ACSL4 and COX2 expression and the decreased GPX4 and FTH1 expression by H/R in HK-2 cells, while this trend was reversed by Exo isolated from USCs transfected with sh-TUG1 (Fig. 5J). These results suggested that lncRNA TUG1 carried by USC-Exo suppresses H/R-induced ferroptosis in HK-2 cells.

### LncRNA TUG1 regulates ACSL4 mRNA stability by interacting with SRSF1

**Fig. 6:**
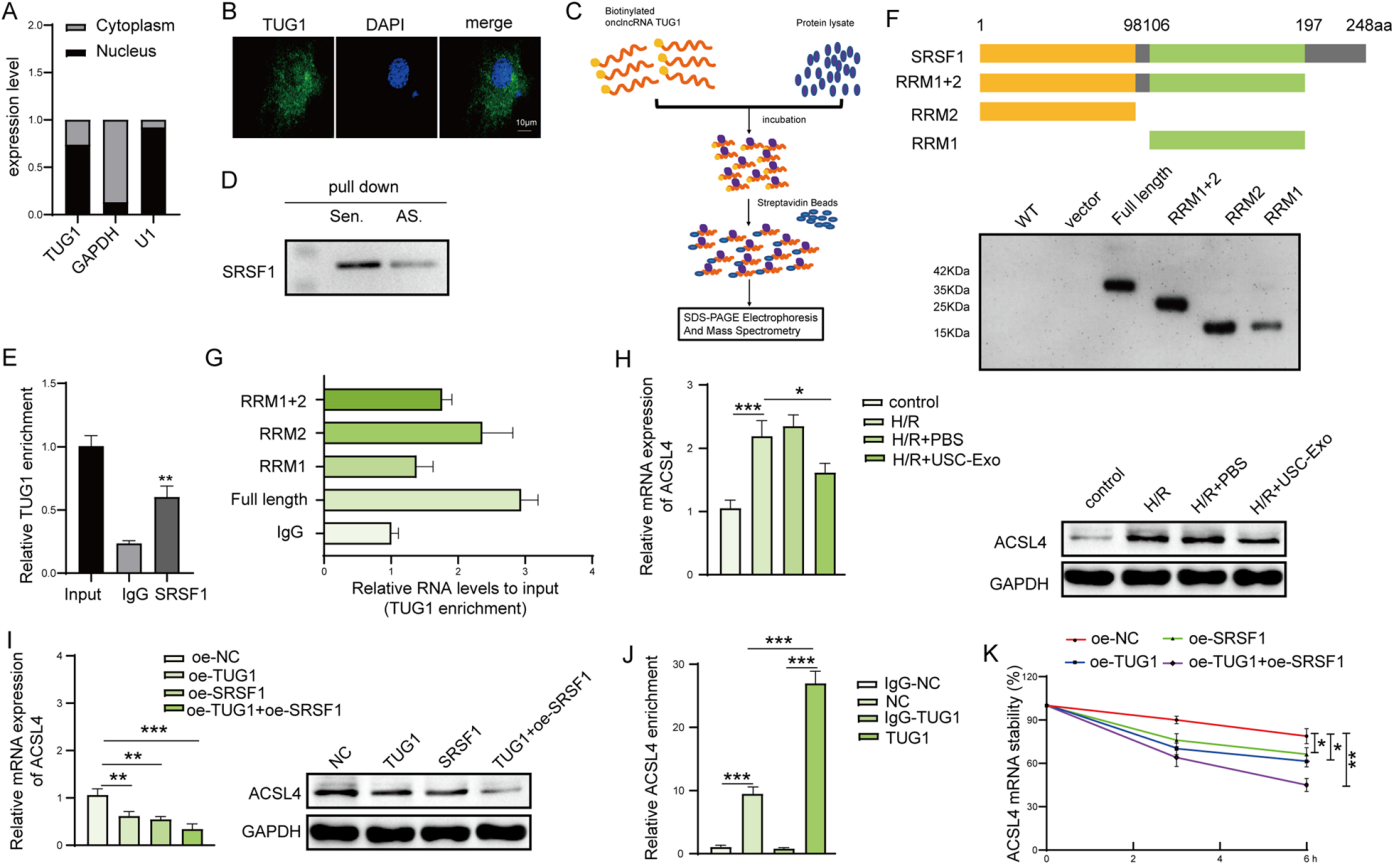
LncRNA TUG1 regulates the stability of mRNA by interacting with SRSF1. **A**, **B** The localization of TUG1 in HK-2 cells was determined by subcellular fractionation assay and RNA FISH assay. Scale bar: 10 µm. **C** The schematic illustration of RNA pull-down assay. Interaction between lncRNA TUG1 and SRSF1 was confirmed by **D** RNA pull-down and **E** RIP assay. **F** Schematic illustration of SRSF1 full-length protein and truncations; their expression in E. coli was shown in the SDS-PAGE gel below. **G** The binding of TUG1 to SRSF1 full-length protein and truncated proteins was assessed by RIP. **H** The effect of USC-Exo on the expression of ACSL4 was checked by qPCR and western blot. **I** The effect of TUG1 and SRSF1 on the ACSL4 expression was assessed by qPCR and western blot. **J** RIP to determine the interaction between ACSL4 mRNA and SRSF1 protein using anti-SRSF1 or IgG (negative control) in HK2 cells in the presence or absence of TUG1 overexpression. **K** The effect of TUG1 and SRSF1 on the stability of ACSL4 mRNA was measured by qPCR. *N* = 3 samples per groups. *p < 0.05, **p < 0.01, ***p < 0.001. The above data were all measurement data and expressed as means ± SD. All experiment was performed three times. Sen., sense; As., antisense

Subcellular fractionation analysis and FISH assay showed TUG1 was expressed in both cytoplasm and nuclear (Fig. 6A, B). RNA pull-down assay was processed as shown in Fig. 6C, and the results demonstrated that SRSF1 was existed in the samples of pull-down, which suggested that TUG1 was interacted with SRSF1 (Fig. 6D). In addition, SRSF1 antibody cached more TUG1 compared with IgG (Fig. 6E). To determine which location of SRSF1 is interacted with lncRNA TUG1, we designed and purified SRSF1 full-length protein and some protein fragments, like RRM1, RRM2, and C terminal structural domain. We found that all three protein domains were involved in the interaction between TUG1 and SRSF1 (Fig. 6F, G). Additionally, USC-Exo significantly suppressed H/R-induced ACSL4 mRNA and protein expression (Fig. 6H). Furthermore, TUG1 and SRSF1 treatment significantly downregulated ACSL4 mRNA and protein expression (Fig. 6I). Next, RIP assay showed ACSL4 mRNA was enriched by SRSF1 versus control lgG antibody; moreover, this enrichment was significantly increased by TUG1 overexpression (Fig. 6J). Furthermore, TUG1 and SRSF1 could promote the stability of ACSL4 mRNA (Fig. 6K). Therefore, lncRNA TUG1 regulates the stability of ACSL4 mRNA by interacting with SRSF1.

### LncRNA TUG1 suppresses H/R-induced ferroptosis by regulating SRSF1/ACSL4 in HK-2 cells

**Fig. 7:**
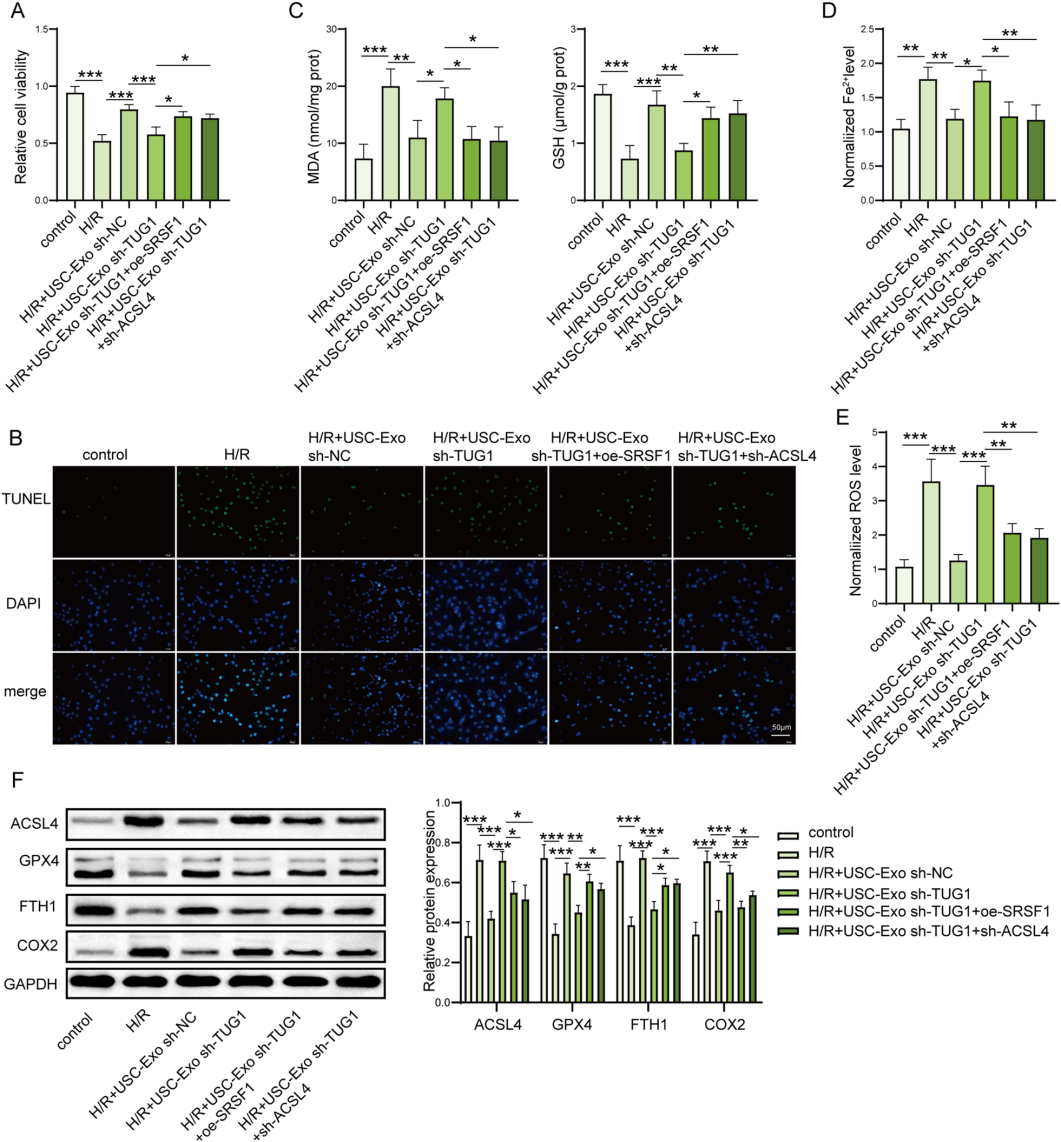
LncRNA TUG1 suppressed H/R-induced ferroptosis in HK-2 cells by regulating SRSF1/ACSL4. **A** Cell viability was assessed by CCK-8 kits. **B** Cell death of HK-2 cells was measured by TUNEL assay. TUNEL-positive cell quantitative bar diagrams are shown. Scale bar: 50 µm. **C** The levels of MDA and GSH in the supernatants of HK-2 cells were detected by commercial kits. **D** The levels of Fe^2+^ in HK-2 cells were checked by commercial kits. **E** The levels of ROS in HK-2 cells were detected using DCFH-DA probe. **F** The protein levels of ACSL4, GPX4, FTH1, and COX2 in HK-2 cells were assessed by western blot and shown as blot image and quantitative bar diagram. *N* = 3 samples per groups. *p < 0.05, **p < 0.01, ***p < 0.001. The above data were all measurement data and expressed as means ± SD. All experiment was performed three times

Then, we determined the inhibition of ferroptosis by lncRNA TUG1 through SRSF1/ACSL4 in HK-2 cells. We found that downregulation of TUG1 by shRNA transfection reversed the protective effects of USC-Exo on the cell viability and death in H/R-treated HK2 cells, while SRSF1 upregulation or ACSL4 downregulation partially reversed these effects of sh-TUG1 on cell viability and death in USC-Exo-treated HK2 cells stimulated by H/R (Fig. 7A, B). The levels of MDA, iron level, and ROS were increased, and GSH was decreased by TUG1 inhibited USC-Exo in USC-Exo-treated HK2 cells stimulated by H/R (Fig. 7C–E), while SRSF1 upregulation or ACSL4 downregulation partially reversed this reduced effect by sh-TUG1-treated USC-Exo. Moreover, the protein expression of ACSL4 and COX2 was decreased and GPX4 and FTH1 were increased in in USC-Exo-treated HK2 cells stimulated by H/R, while TUG1 downregulated USC-Exo markedly reversed those effect by USC-Exo in H/R-treated HK-2 cells (Fig. 7F). And, SRSF1 upregulation or ACSL4 downregulation partially reversed the trend of sh-TUG1-transfected USC-Exo on ferroptosis (Fig. 7F). These data suggest that lncRNA TUG1 ameliorates H/R-induced ferroptosis by regulating SRSF1/ACSL4 in HK-2 cell.

### LncRNA TUG1 ameliorates IRI-induced AKI by suppressing ACSL4-mediated ferroptosis

**Fig. 8:**
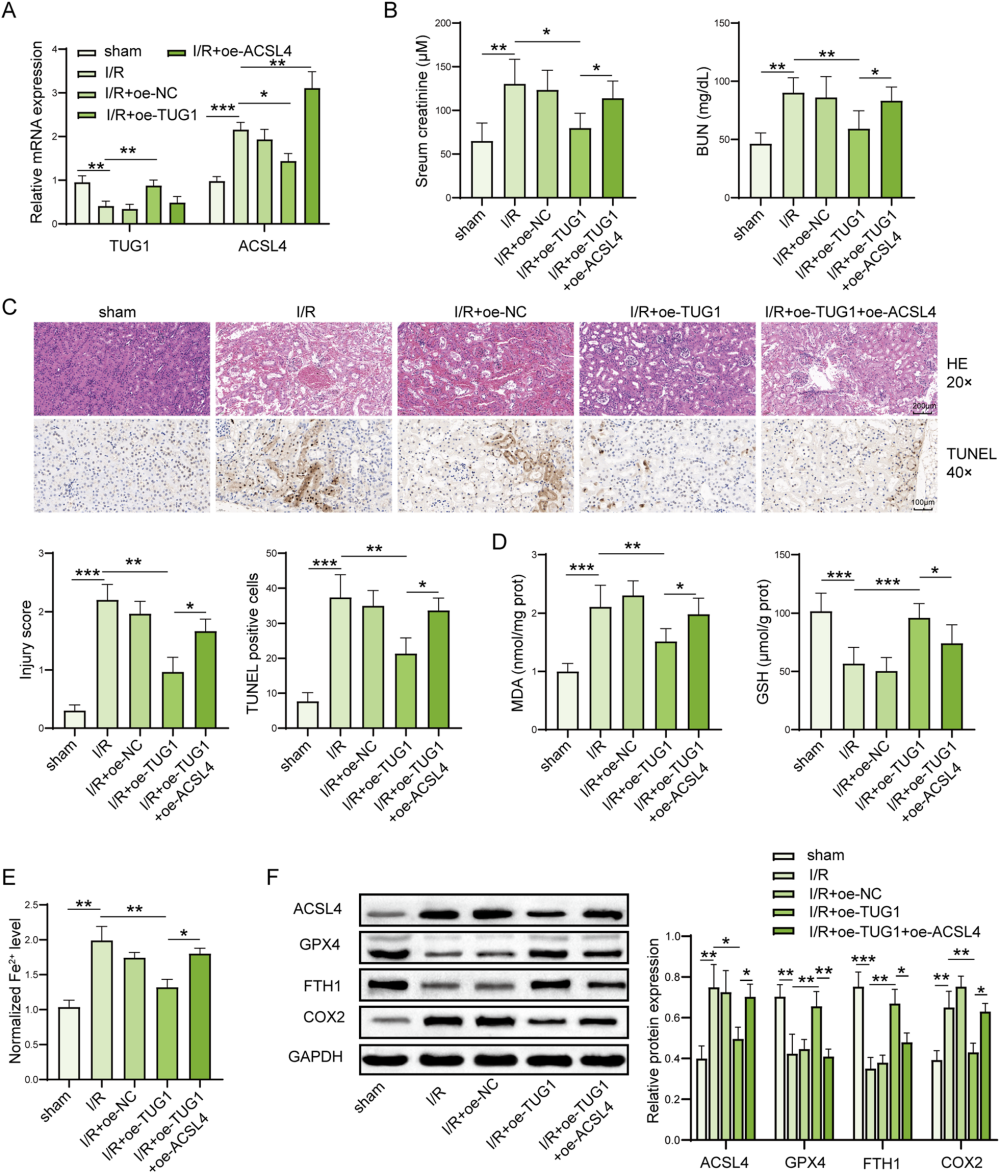
LncRNA TUG1 ameliorates IRI-induced kidney injury by suppressing ACSL4-mediated ferroptosis. **A** The expression of TUG1 and ACSL4 was measured in kidney tissues via qPCR. **B** Serum levels of creatinine and BUN were assessed by colorimetric method. **C** The injury and cell death of kidney tissues shown by HE dyes and TUNEL assay. Representative images of kidney H&E dyes and TUNEL staining was presented, and the kidney injury score and TUNEL-positive cell quantitative bar diagrams are shown. HE, scale bar: 200 µm; TUNEL, scale bar: 100 µm. **D** The levels of MDA and GSH in the mice were assessed by using commercial kits. **E** The levels of Fe^2+^ in the mice were checked by using commercial kits. **F** The protein levels of ACSL4, GPX4, FTH1, and COX2 in the kidney tissues were assessed by western blot and shown as blot image and quantitative bar diagram. *N* = 6 mice per groups. *p < 0.05, **p < 0.01, ***p < 0.001. The above data were all measurement data and expressed as means ± SD

**Fig. 9:**
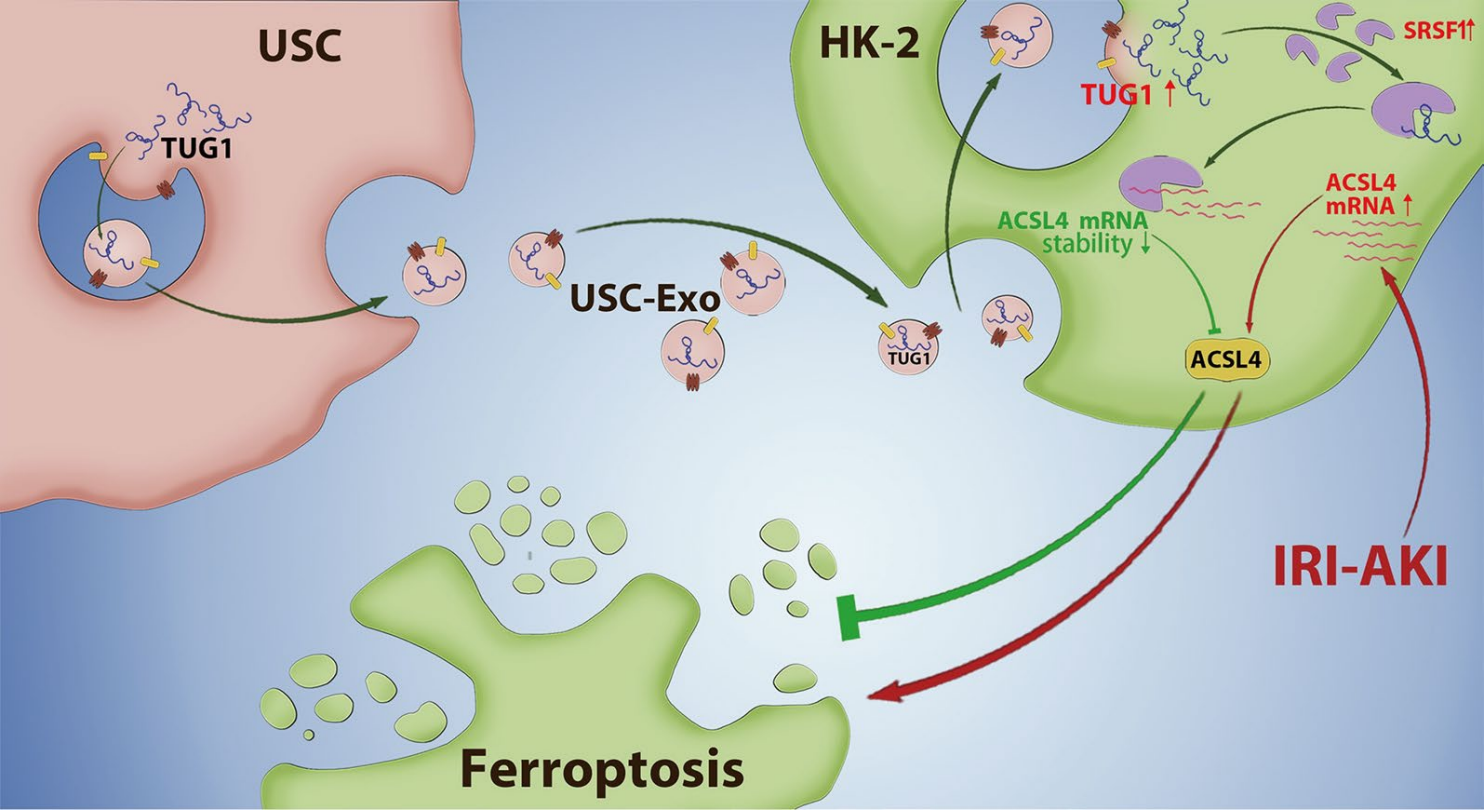
USC-Exo ingested by kidney cells upregulate lncRNA TUG1 expression, which promotes ACSL4 mRNA decay by interacting with SRSF1, then suppresses ACSL4-mediated cell ferroptosis, and furtherly improves IRI-induced AKI

Finally, the in vivo impact of lncRNA TUG1 on ferroptosis and kidney injury was assessed and we adopted plasmids tool to transiently overexpress TUG1 and ACSL4 expression in mice. We checked the efficiency of plasmids in the mice kidney tissue by qPCR. The expression of TUG1 or ACSL4 was upregulated in the mice kidney tissues after transfected by TUG1 and ACSL4 plasmids, respectively (Fig. 8A). We found that lncRNA TUG1 treatment significantly reduced levels of serum creatinine and BUN induced by IRI-induced AKI mice (Fig. 8B). The morphological tubular HE and TUNEL dye also showed that lncRNA TUG1 treatment significantly alleviated IRI-induced kidney injury evidenced by decreased kidney injury score and inhibited kidney cell death (Fig. 8C). However, ACSL4 overexpression partially reversed this protective effect on kidney injury by lncRNA TUG1 (Fig. 8B, C). We also found that the levels of MDA and iron were significantly increased and GSH level was remarkably decreased in IRI-induced AKI mice compared to the sham mice, while lncRNA TUG1 markedly decreased those in IRI-induced AKI mice (Fig. 8D, E). Moreover, ACSL4 partially reversed this reduced effect by lncRNA TUG1. Additionally, the protein expression of ACSL4 and COX2 was increased, and GPX4 and FTH1 were decreased in IRI-induced AKI mice compared to the sham mice, while lncRNA TUG1 markedly reversed those in IRI-induced AKI mice (Fig. 8F). And, ACSL4 upregulation partially reversed the inhibition effect on ferroptosis by lncRNA TUG1 (Fig. 8F). Taken together, lncRNA TUG1 ameliorates IRI-induced kidney injury by suppressing ACSL4-mediated ferroptosis (Fig. 9).

## Discussion

As we known, human USC treatment has been proved to present protective effects on in ischemic AKI model [9], and convincing evidence indicates that extracellular vesicles like exosomes secreted by stem cells could improve AKI [29]. In line with the previous studies, we found that USC-Exo treatment showed a beneficial effect on IRI-induced AKI with evidence of decreased BUN and serum creatinine levels and reduced cell death of kidney. However, the mechanism of the protective effect of USC-Exo on IRI-induced AKI is not fully clarified. In the present study, we investigated the mechanisms underlying the protective effect of USC-Exo on IRI-induced AKI by focusing on the potential involvement and modulation of ACSL4-mediated ferroptosis. We firstly demonstrated a novel mechanism underlying the protective role of USC-Exo rich in lncRNA TUG1 against IRI-induced AKI, which was involved in inhibiting ACSL4-mediated ferroptosis through interacting with SRSF1.

Ferroptosis was recently discovered as a regulated cell death which is induced by the accumulation of iron-mediated lipid peroxidation, and subsequent plasma membrane ruptures and the release of damage-associated molecules [18]. Multiple regulators of iron metabolism related to iron uptake, storage, and utilization may impact the process of ferroptosis, such as transferrin/transferrin receptor, and iron-storage protein ferritin includes ferritin light chain (FTL)/ferritin heavy chain 1 (FTH1), heme oxygenase 1, and so on [30, 31]. As lipid peroxidation is another key factor for driving ferroptotic cell death, many oxidative and antioxidant pathways involved in the regulation of cell ferroptosis, such as COX2, ACSL4, GSH, and GPX4 [19, 32]. In the present study, the most common biomarkers of ferroptosis were used in current research: cellular Iron levels, product of lipid peroxidation like MDA, GSH levels, ACSL4, GPX4, upregulation of PTGS2 gene like COXC2, and so on [33]. We found that IRI increased the expressions of MDA, iron, ROS, ACSL4, and COX2 and decreased GSH, GPX4, and FTH1 in the kidney tissues. These data suggested that ferroptosis was upregulated in IRI-induced AKI. Convincing evidence proved that strategies like iron chelation therapy, targeting iron metabolism-related proteins, and direct inhibitors of ferroptosis may provide new therapeutic methods for AKI [34, 35]. In our experiments, it is interesting that USC-Exo treatment had the same protective effect as ferroptosis inhibitor Fer-1 in IRI-induced kidney injury. Together, we conclude that USC-Exo treatment suppresses kidney cell ferroptosis in vitro and in vivo and has a protective effect on IRI-induced kidney injury.

Exosomes are vesicles with a bilayer membrane structure, which are rich in proteins, lipids, miRNAs, lncRNA, and other RNA species [12]. Increasing evidence suggests that lncRNA carried in exosomes may have a vital role in the immune response of pathology of diseases and represent a novel target for AKI. It has been reported that lncRNA TUG1 of kidney tissue was downregulated in LPS-induced and IRI-induced AKI. At the same time, upregulating TUG1 presented a protective effect on IRI-induced kidney injury [17, 18]. Chen et al. have demonstrated that overexpression of TUG1 significantly alleviated kidney injury and cell apoptosis by targeting miR-494-3p/E-cadherin in I/R-induced AKI [17]. During our experiments, we observed that the levels of lncRNA TUG1 were significantly increased in USC-Exo. Thus, we speculated that lncRNA TUG1 might participate in the protective effect of USC-Exo on IRI-induced AKI for further investigation. To investigate the role of lncRNA TUG1 carried by USC-Exo in the regulation of cell ferroptosis, we adopted shRNA tool to transiently knockdown TUG1 expression in USC-Exo. Consistent with our speculation, further experiments confirmed that sh-TUG1 gene-silenced USC-Exo showed lower TUG1 expression and reversed the protective effects on cell ferroptosis by sh-NC USC-Exo treatment in H/R-treated HK-2 cells. Moreover, consistent with previous study, we demonstrated that upregulating TUG1 expression by plasmids transfection inhibited cell ferroptosis and improved kidney injury induce by IRI in vivo. Therefore, these results suggested that USC-Exo suppresses H/R-induced ferroptosis by transferring lncRNA TUG1 in HK-2 cells.

However, the molecular mechanism for suppressing ferroptosis by lncRNA TUG1 carried by USC-Exo remains unclear. It has shown that lncRNA regulates the stability and expression of downstream target mRNA and participates in the process of disease by interacting with RNA-binding proteins [23]. SRSF1 is the archetype member of the SR protein family of splicing regulators, which has been involved in several key aspects of mRNA metabolism, such as mRNA splicing, stability, and translation, as well as other mRNA-independent processes [24]. Studies have shown that SRSF1 expression is downregulated in myocardial IRI, and its overexpression can inhibit myocardial cell apoptosis [25]. Acyl-CoA synthetase long-chain family member 4 (ACSL4), an enzyme involved in fatty acid metabolism, is considered as a specific biomarker and driver of ferroptosis [20]. Silent ACSL4 gene reduces cell ferroptosis and protects the brain and gut IRI [21, 22]. In present study, we predicted that RNA-binding protein SRSF1 had a potential binding relationship with lncRNA TUG1 and ACSL4 through an online bioinformatics tool-Star Base. Therefore, we hypothesized that lncRNA TUG1 might regulate ACSL4 expression by interacting with SRSF1. Here, we firstly proved that lncRNA TUG1 was interacted with SRSF1 evidenced by RNA pull-down assay and RIP assay. We also proved that lncRNA TUG1 regulates the stability of mRNA ACSL4 by interacting with SRSF1. In addition, downregulation of TUG1 by shRNA transfection reversed the protective effects of USC-Exo on ferroptosis in H/R-treated HK2 cells, while SRSF1 upregulation or ACSL4 downregulation partially reversed these effects of sh-TUG1 USC-Exo on cell ferroptosis in HK2 cells stimulated by H/R. Therefore, we firstly demonstrated that lncRNA TUG1 suppressed ACSL4-mediated ferroptosis by interacting with SRSF1 in HK-2 cells.

## Conclusions

In conclusion, we found that lncRNA TUG1 was carried by USC-Exo downregulation of ACSL4 expression in kidney cells by interacting with SRSF1, then inhibited ACSL4-mediated cell ferroptosis, and thus improved kidney injury in IRI-induced AKI. This study revealed a novel mechanism by which lncRNA TUG1 carried USC-Exo protected IRI-induced AKI by inhibiting cell ferroptosis and suggested that the reprogramming of iron homeostasis is essential in renal injury, and USC-Exo and TUG1 overexpression is a promising therapeutic method for the treatment of IRI-AKI.

## Data Availability

All data are available from the first author (Zejia Sun) on reasonable request.

## Abbreviations

ACSL4: Acyl-CoA synthetase long-chain family member 4
AKI: Acute kidney injury
BUN: Blood urea nitrogen
DMEM: Dulbecco's Modified Eagle Medium
FISH: Fluorescence in situ hybridization
GSH: Glutathione
HK-2: Human proximal tubular epithelial cells
IRI: Ischemic/reperfusion injury
MDA: Malondialdehyde
RIP: RNA immunoprecipitation
ROS: Reactive oxygen species
Scr: Serum creatinine
SRSF1: Serine/arginine splicing factor 1
TEM: Transmission electron microscopy
TUG1: Taurine-upregulated gene 1
TUNEL: Transferase terminal UTP nick-end labeling
USCs: Urine-derived stem cells
